# The Combined Effects of Body Weight Support and Gait Speed on Gait Related Muscle Activity: A Comparison between Walking in the Lokomat Exoskeleton and Regular Treadmill Walking

**DOI:** 10.1371/journal.pone.0107323

**Published:** 2014-09-16

**Authors:** Klaske Van Kammen, Annemarijke Boonstra, Heleen Reinders-Messelink, Rob den Otter

**Affiliations:** 1 Center for Human Movement Sciences, University of Groningen, Groningen, The Netherlands; 2 Rehabilitation Center ‘Revalidatie Friesland’, Beetsterzwaag, The Netherlands; Purdue University, United States of America

## Abstract

**Background:**

For the development of specialized training protocols for robot assisted gait training, it is important to understand how the use of exoskeletons alters locomotor task demands, and how the nature and magnitude of these changes depend on training parameters. Therefore, the present study assessed the combined effects of gait speed and body weight support (BWS) on muscle activity, and compared these between treadmill walking and walking in the Lokomat exoskeleton.

**Methods:**

Ten healthy participants walked on a treadmill and in the Lokomat, with varying levels of BWS (0% and 50% of the participants’ body weight) and gait speed (0.8, 1.8, and 2.8 km/h), while temporal step characteristics and muscle activity from Erector Spinae, Gluteus Medius, Vastus Lateralis, Biceps Femoris, Gastrocnemius Medialis, and Tibialis Anterior muscles were recorded.

**Results:**

The temporal structure of the stepping pattern was altered when participants walked in the Lokomat or when BWS was provided (i.e. the relative duration of the double support phase was reduced, and the single support phase prolonged), but these differences normalized as gait speed increased. Alternations in muscle activity were characterized by complex interactions between walking conditions and training parameters: Differences between treadmill walking and walking in the exoskeleton were most prominent at low gait speeds, and speed effects were attenuated when BWS was provided.

**Conclusion:**

Walking in the Lokomat exoskeleton without movement guidance alters the temporal step regulation and the neuromuscular control of walking, although the nature and magnitude of these effects depend on complex interactions with gait speed and BWS. If normative neuromuscular control of gait is targeted during training, it is recommended that very low speeds and high levels of BWS should be avoided when possible.

## Introduction

The ability to walk is a key aspect of independent functioning, and as such it represents an important rehabilitation goal for persons with reduced ambulatory skills [1]. The re-learning of gait movements involves the development of relatively stable changes in spinal and supra-spinal networks that, in order to be functionally useful, need to be shaped by task-specific sensory (e.g. proprioceptive, somatosensory) information [2]. In line with this notion, studies on the effectiveness of locomotor training have concluded that gait rehabilitation strategies need to focus on intensive training of the integral locomotor task [3]–[5], and should thus involve the production of stepping movements with a high number of movement repetitions. Robot Assisted Gait Training (RAGT) implements the above-mentioned principles, by combining body weight supported treadmill training with actuated exoskeletons to provide (semi-) automated training. In RAGT, locomotor task constraints (e.g. support, propulsion, stability, and foot clearance) can be simplified by providing body weight support (BWS) and movement guidance, so that patients who are unable to voluntarily accommodate these constraints can still be exposed to the task-specific sensory information necessary for the re-learning of gait [6]. Implied in the use of actuated exoskeletons for gait training is that, compared to manually assisted training, the parameter space that is available to physically affect the gait pattern is reduced to three dimensions: treadmill speed, the level of BWS, and the level of movement guidance provided by the exoskeleton. The reduced parameter space in RAGT necessitates the development of specialized protocols to fully exploit the motor learning potential that this type of training has to offer. The development of such protocols should be firmly grounded in knowledge on locomotor control and motor learning, and requires insight into how training parameters alter locomotor task demands and locomotor control.

A unique aspect of RAGT is the use of the exoskeleton to provide the supportive force field (or ‘guidance’) that guides the legs through the gait cycle. The level of guidance that is offered by the actuated exoskeleton can be adjusted to the specific needs of the patient and, depending on the specific locomotor capabilities of the patient, can be reduced to nil allowing free exploration of coordinative possibilities under safe conditions. However, even when the exoskeleton is not actuated to provide guidance, the implied mechanical coupling between leg and skeleton movements may alter task constraints and the sensory consequences of voluntary leg movements. First, the exoskeleton imposes impedance to the limbs, which can potentially slow down movements of leg segments [7]. Whereas during unrestricted walking, the swinging leg acts as a pendulum at frequencies approaching its natural frequency [8], adding impedance to the leg may alter swing and stride time [9], necessitating adaptations in neuromuscular control [9]–[10]. Second, movements of the exoskeleton are restricted to the sagittal plane, thus reducing the degrees of freedom available to perform the locomotor task. Since movements in the frontal and transversal plane are prominent during gait [11]–[12], these restrictions potentially alter the task constraints under which locomotor control operates naturally. Clearly, these altered task constraints and their effect on locomotor control should be considered when designing training protocols for RAGT.

For a good understanding of how training conditions typical of RAGT affect locomotor control, it is important to simultaneously address all training parameters and assess their mutual interactions. Because mechanical impedance imposed upon the leg naturally depends on segment velocity, the effects of the exoskeleton are likely to depend on gait speed and should therefore not be studied in isolation. Similarly, although the speed of progression is an important determinant of spatial and temporal step characteristics [8], the relationship between speed and step characteristics is modulated by the amount of BWS that is provided [13]. To understand how these combined parameters alter the neuromuscular control of walking it is important to establish the effects of body weight support and treadmill speed on gait related muscle activity and compare these between exoskeleton walking and regular treadmill walking. Previous research on muscle activation in exoskeletons has focused mainly on the Lokomat, a commercially available and widely used device for RAGT [14]–[15]. Results obtained in the Lokomat have shown that the global patterning that characterizes the synergistic neuromuscular control of unrestrained walking is unaffected by the exoskeleton, regardless of treadmill speed [16]–[17]. However, at the level of individual muscles, local alterations in the amplitude of muscle activation are apparent, e.g. the activity of quadriceps and hamstrings is increased whereas, the activity of ankle extensors and flexors is decreased in the Lokomat exoskeleton [18]–[19]. Although these results are important for understanding how actuated exoskeletons alter neuromuscular control and what remains stable, so far the analyses have been restricted to the main effects of individual training parameters. A notable exception is the study by Hidler and Wall [18] who failed to find interactions between type of walking (Lokomat exoskeleton vs treadmill walking) and gait speed, although the range of gait speeds studied was rather small.

The aim of the present study was to obtain a more complete account of the effects of training parameters involved in RAGT on the neuromuscular control of walking. To this end, we systematically assessed the effects of BWS and treadmill speed, as well as their mutual interactions, on temporal step parameters and muscle activity, and compared these between regular treadmill walking and walking in the Lokomat exoskeleton.

## Methods

### Participants

Ten healthy participants (6 females, age 20.9+/−2.2 yrs, mean body height 1.82+/−0.04 meters, mean body weight 77.90+/−9.6 kilograms) volunteered for this study. Participants did not suffer from any disorder that is known to affect gait, balance or muscle activity.

### Ethics statement

The procedures of this study were approved by the Medical Ethical Committee of the University Medical Center Groningen, the Netherlands, and were in accordance with the principles outlined in the Declaration of Helsinki [20]. All participants gave their written informed consent.

### Materials

#### The exoskeleton

The Lokomat Pro version 6.0 (*Hocoma AG, Volketswil, Switzerland*) was used for walking trials in the exoskeleton. The Lokomat is a bilaterally driven gait orthosis that is combined with a body-weight support system and a treadmill [15]. The orthosis moves the legs along a specified trajectory in the sagittal plane, with hip and knee joints of the orthosis actuated by linear drives that are integrated into an exoskeleton. A so called ‘path control’ algorithm is used to guide the legs of the user through a haptic tunnel. An impedance controller supplies a supportive force field and gently corrects leg movements towards the specified trajectory when necessary. The level of impedance can be controlled, so that the extent to which users can actively move their legs along the haptic tunnel, can be varied systematically. Since the present experiment focused on differences between walking conditions (exoskeleton vs treadmill walking) in the context of different settings for treadmill speed and BWS, the amount of movement guidance was set to zero. This allowed a clean experimental assessment of the combined effects of BWS and treadmill speed and how these effects are modulated in the exoskeleton. In this ‘free run’ mode, the impedance that determines the contribution of the driven orthosis to leg movements is set to zero, providing a walking condition in the Lokomat in which full range leg movements are possible, and as such most closely mimics unrestrained walking. Also, in this mode compensatory torques are generated to compensate interaction forces between exoskeleton and user that result from inertia of the exoskeleton, gravity and friction. This largely reduces, but not completely eliminates, the interaction torques [21]. Trials outside the exoskeleton (‘treadmill walking’) were conducted on the same treadmill, but participants were disengaged from the exoskeleton.

#### Electromyography and detection of gait events

Signals were pre-amplified and A/D converted (22 bits) using a 32-channel Porti7 portable recording system (*Twente Medical Systems, Enschede, The Netherlands*). The system has a common mode rejection >90 dB, a 2 µVpp noise level and an input impedance >1 GV. As in similar gait studies (e.g. [16]), EMG signals were sampled at 2048 Hz, which is adequate to capture the relevant frequency content of the EMG, and allows for detection of foot contact times at a high temporal resolution. Before sampling, incoming EMG signals were filtered using a 10 Hz fourth order Butterworth high-pass filter, to attenuate movement artefacts. Signals were fed from the portable unit to a laptop computer for storage and offline analysis.

Self-adhesive, disposable Ag/AgCl electrodes (*Kendall/Tyco ARBO; Warren, MI, USA*) with a 25 mm diameter and a minimum electrode distance of 25 mm, were used to record activity from the following muscles, in the right leg: (1) Erector Spinae (ES), (2) Gluteus Medius (GM), (3) Vastus Lateralis (VL), (4) Biceps Femoris (BF), (5) Medial Gastrocnemius (MG) and (6) Tibialis Anterior (TA). To improve skin conduction, the skin was abraded and cleaned with alcohol, and body hair was removed at the electrode sites. Electrode placement was in accordance with SENIAM conventions [22].

To detect gait events, customized insoles (Pedag international VIVA) containing four pressure sensors (FSR402, diameter 18 mm, loading 10–1000 g; one under the heel, 3 under the forefoot), were placed in the footwear of participants. Signals from these sensors were fed to one of the analogue inputs of the EMG amplifier, sampled at 2048 Hz, and stored on the laptop computer for further processing.

### Procedure

Prior to the experiment, individual adjustments were made to the exoskeleton to suit the anthropometric characteristics of the participant. Hip width, length of upper and lower leg, size and position of the leg cuffs were adjusted to assure that walking in the Lokomat was as natural and comfortable as possible. Although the Lokomat allows fixation of the ankle joints by means of elastic foot lifters, these were not used to allow free ankle movements and provide an adequate comparison with treadmill walking. Participants walked on their own foot wear.

Participants walked a total of 12 trials, with each trial representing a unique combination of walking condition (treadmill or Lokomat), BWS and gait speed. Dynamic BWS was provided using a suspended harness and was adjusted to support 0% or 50% of the participants’ body weight. This type of support allows free vertical movement within a certain range, while the level of weight support within this range is held approximately constant. The 50% BWS was chosen because this approximately represents the maximal amount of support that is provided to patients during training [23], [18]. Gait speed was controlled by varying the treadmill speed, and was set to 0.8, 1.8 and 2.8 km/h. These speeds cover most of the possible speed range of the Lokomat which ranges from 0.5 to 3.2 km/h. In both gait conditions (treadmill and Lokomat), participants were required to complete (2 levels of BWS×3 gait speeds  = ) 6 trials. To avoid possible after-effects of the Lokomat, all participants were first assessed during treadmill walking. Trials within each gait condition were randomized over participants to prevent order effects.

At the start of each trial, participants were allowed practice time to get familiar to the specific setting of the treadmill or Lokomat, until he/she indicated to be comfortable, and recording commenced. To obtain an approximately equal number of strides per trial, the duration of measurements depended on gait speed and lasted 120, 70, and 40 seconds, at 0.8, 1.8, 2.8 km/h, respectively.

### Data analysis

#### Signal analysis

Offline analysis of EMG and foot sensor data was performed using custom-made software routines in Matlab (version 2011a; *The Mathworks Inc., Natick, MA*). Using the foot-sensor data, four sub-phases of the gait cycle were distinguished: The first double support (DS1), the single support (SS), the second double support (DS2) and the swing (SW) phase. Step phase durations were analyzed to assess the effects of walking condition, gait speed, and BWS on the temporal structure of the stepping pattern. The EMG data were full wave rectified and low-pass filtered using a zero lag fourth order Butterworth filter with a 20 Hz cutoff. The data were time normalized with respect to gait cycle time (from heelstrike to heelstrike), and amplitude normalized with respect to the maximum amplitude over all conditions, for each participant. To allow statistical comparison between walking conditions, the amplitude-normalized data were summed for each of the four sub-phases (DS1, SS, DS2 and SW) and averaged over strides, for each participant and each condition.

#### Statistical analysis

To compare step phase durations and levels of muscle activity between gait conditions, a series of three-way univariate repeated measures ANOVA’s were used, testing the effects of the factors Speed (0.8 vs 1.8 vs 2.8 km/h), BWS (0% vs 50%), and Walking Condition (walking in the Lokomat vs treadmill walking), for each of the four sub-phases (DS1, SS, DS2 and SW), separately. This procedure was used to test simultaneously for all main effects of the above-mentioned factors, as well as their 2-way and 3-way interactions. Because temporal symmetry was assumed for the present group of participants, the analysis of step phase durations was restricted to the DS1 and SS phases. Main effects and all two way and three way interactions were evaluated using an alpha level of 0.05. When a factor A showed a significant main effect and was also involved in a significant interaction with another factor B, the interpretation of the main effect of A was not straightforward. To determine whether main effects in this specific situation were meaningful, simple main effects of A were analyzed for each level of factor B. A main effect for factor A was considered meaningful only if (1) significant main effects could be determined for each level of factor B, and (2) the effects of the simple main effects of A were in the same direction for all levels of B.

The Benjamini-Hochberg procedure was applied to the test results to control the false discovery rate and correct for multiple testing [24]. All statistical processing was done in SPSS version 19 for Windows (*SPSS,Chicago, IL, USA*).

The data that were collected and reported about in the manuscript have now been made publicly available on DataDryad. The digital object identifier for our data is: doi:10.5061/dryad.22c78.

## Results

In a number of instances, a factor was simultaneously involved in a significant main effect and one or more interactions. In these cases, main effects are discussed here only if the analysis of simple main effects indicated that they were meaningful (see ‘Statistical analysis’). In other cases, discussion of the effects will be restricted here to the interactions. However, for a complete overview of all results from the repeated measures ANOVA, we refer the reader to Tables 1 and 2. For both step phase durations and muscle activity parameters, no significant three-way interactions were found, so they will not be discussed here.

**Table 1:** Overview of the results from univariate testing of the effects of Condition (treadmill vs Lokomat exoskeleton), Speed (0.8, 1.8 and 2.8 km/h), and BWS (0 and 50% of body weight), and two-way interactions, on step phase durations.

	Condition		Speed		BWS		Condition×Speed		Condition×BWS		Speed×BWS	
	F(1,9)	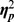	F(2,18)	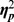	F(1,9)	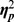	F(2,18)	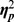	F(1,9)	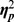	F(2,18)	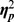
**Step Phase Duration**												
DS1	52.09***	0.85	23.66**	0.72	44.62***	0.83	55.56***	0.86	-	-	-	-
SS	16.32**	0.64	21.33**	0.70	26.48**	0.75	-	-	8.83*	0.50	-	-

* = p<.05;

** = p<.01;

***p<.001;

*- = not significant.*

**Table 2:** Overview of the results from univariate testing of the effects of Condition (treadmill vs Lokomat exoskeleton), Speed (0.8, 1.8 and 2.8 km/h), and BWS (0 and 50% of body weight), and two-way interactions, on muscle activity during the phases of gait.

	Condition			Speed		BWS		Condition×Speed		Condition×BWS		Speed×BWS	
	F(1,9)	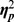		F(2,18)	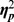	F(1,9)	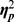	F(2,18)	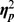	F(1,9)	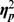	F(2,18)	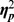
**Erector Spinae**													
DS1	7.64*	0.46		-	-	-	-	-	-	-	-	-	-
SS	10.91**	0.55		5.30*	0.37	-	-	14.18**	0.61	-	-	-	-
DS2	7.00*	0.44		-	-	-	-	-	-	27.23**	0.75	8.88**	0.50
SW	7.54*	0.46		6.53**	0.42	-	-	7.02**	0.44	-	-	-	-
**Gluteus Medius**													
DS1	-	-		26.03***	0.74	-	-	-	-	-	-	-	-
SS	-	-		8.53**	0.49	10.36*	0.54	6.24**	0.41	-	-	-	-
DS2	**-**	**-**		-	-	-	-	-	-	-	-	-	-
SW	**-**	**-**		-	-	-	-	-	-	-	-	-	-
**Biceps Femoris**													
DS1	-	-		-	-	-	-	-	-	-	-	-	-
SS	27.13**	0.75		8.43**	0.48	19.73**	0.69	10.75**	0.54	-	-	4.84*	0.35
DS2	16.45**	0.65		-	-	-	-	-	-	-	-	-	-
SW	-	-		49.63***	0.85	-	-	-	-	-	-	7.45**	0.45
**Vastus Lateralis**													
DS1	-	-		13.13***	0.59	21.18**	0.70	-	-	8.03*	0.47	8.58**	0.49
SS	7.84*	0.47		-	-	-	-	-	-	9.65*	0.52	-	-
DS2	-	-		-	-	-	-	-	-	11.24**	0.56	-	-
SW	12.31**	0.58		-	-	-	-	-	-	11.53**	0.56	6.72**	0.43
**Gastrocnemoius Medialis**													
DS1	-	-		4.31*	0.32	-	-	-	-	-	-	-	-
SS	-	-		29.27***	0.77	81.79***	0.90	-	-	-	-	7.59**	0.46
DS2	-	-		-	-	25.15**	0.74	-	-	-	-	7.42**	0.45
SW	-	-		7.83**	0.47	-	-	-	-	-	-	-	-
**Tibialis Anterior**													
DS1	18.49**		0.67	9.04**	0.50	-	-	-	-	21.05**	0.70	-	-
SS	10.02*		0.53	4.13*	0.32	-	-	10.59**	0.54	-	-	-	-
DS2	-		-	21.81***	0.71	-	-	7.61**	0.46	-	-	-	-
SW	-		-	30.77***	0.78	-	-	6.67*	0.43	-	-	-	-

* = p<.05; ** = p<.01; ***p<.001; - = not significant.

### Step phase durations

**Figure 1:**
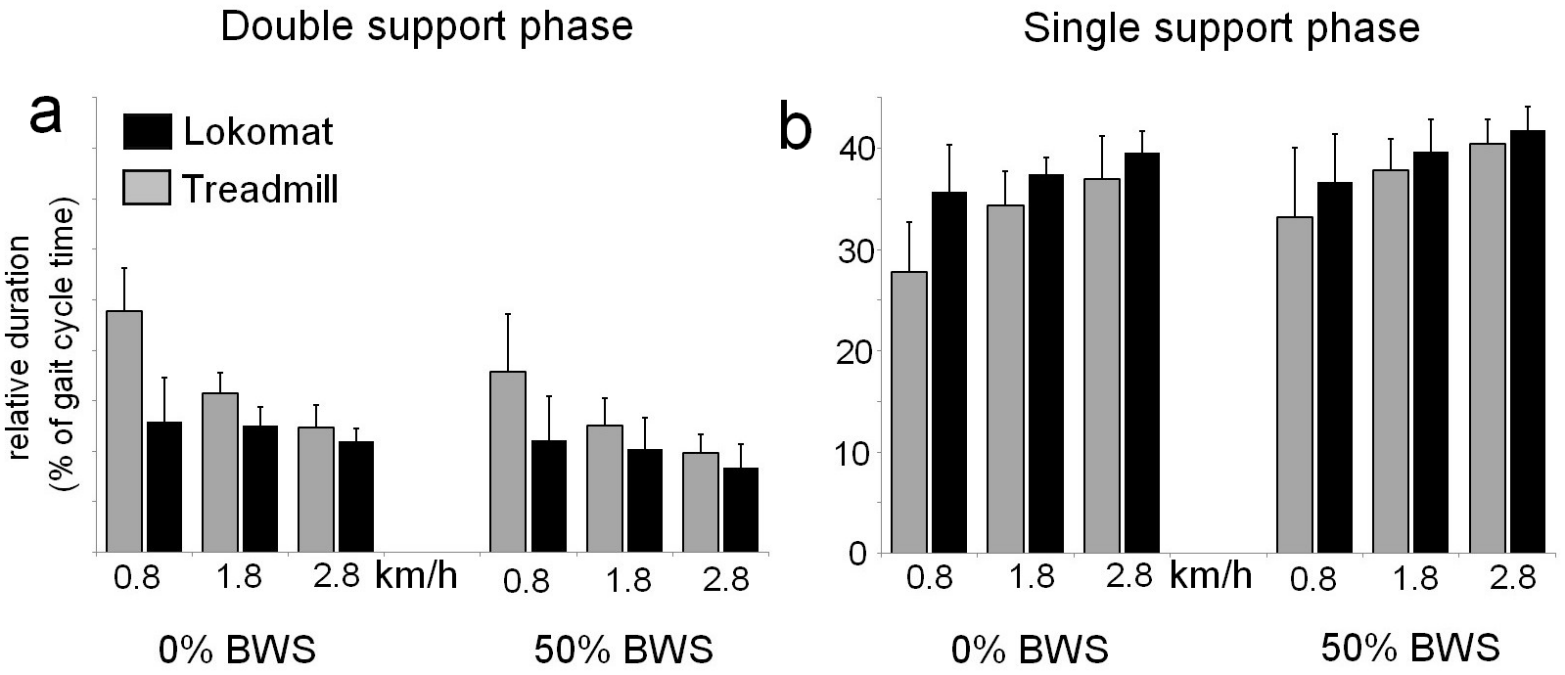
Mean duration of step phases. The mean relative duration (+ standard deviations) of (A) the double support phase and (B) the single support phase, expressed as a percentage of the total gait cycle duration (DS1: first double support phase; SS: single support phase; DS2: second double support phase; SW: swing phase).

Because we assumed symmetry in the present group of participants, only the relative durations of the first double support phase (DS1, equal to contralateral DS2) and the single support phase (SS, equal to contralateral SW) were tested. The mean relative durations of these phases and their associated standard deviations (sd’s) are shown in Figure 1. The results of the statistical tests (i.e. F-values, eta-squared and the level of significance) are summarized in Table 1.

A significant main effect of BWS indicated that supporting 50% of the subject’s body weight resulted in a decrease in DS1 duration compared to the full weight bearing condition (14.7% of gait cycle time vs 11.7%). Similarly, a main effect for the factor Speed revealed that increases in treadmill speed resulted in a systematic shortening of the DS1 phase (16.5% vs 10.4% at 0.8 and 2.8 km/h, respectively). However, the magnitude of this Speed effect depended on walking condition, as indicated by a significant Speed by Condition interaction. Whereas during treadmill walking the relative duration of the DS1 phase was substantially shortened at higher speeds (20.9% at 0.8 km/h vs 11.1% at 2.8 km/h), this speed effect was less pronounced when walking in the exoskeleton (12.1% vs 9.7%).

A main effect of BWS indicated that the support of body weight resulted in an increase in SS duration (35.3% vs 38.3%). Similarly, a main effect of Condition showed that the relative SS durations were longer in the exoskeleton compared to treadmill walking (38.5% vs 35.1%). However, because provision of BWS resulted in lengthening of the SS phase during treadmill walking (see figure 1), differences between walking conditions were attenuated when BWS was provided, as indicated by a significant Condition by BWS interaction. Whereas the mean difference between exoskeleton and treadmill walking was 4.6% in the full weight bearing condition, this was reduced to 2.2% when BWS was provided. Finally, a main effect of Speed showed that, irrespective of BWS and walking condition, longer SS durations were observed at higher speeds (33.3% vs 39.7% at 0.8 and 2.8 km/h).

### Muscle activity

The global patterning of muscle activity remained relatively stable over experimental conditions, although alterations in speed, BWS and walking condition resulted in local changes in the amplitude of muscle output. The results of the statistical tests (i.e. F-values, eta-squared and the level of significance) are summarized in Table 2. Below, the appropriate effects are discussed in more detail.

#### Erector Spinae (ES)

**Figure 2:**
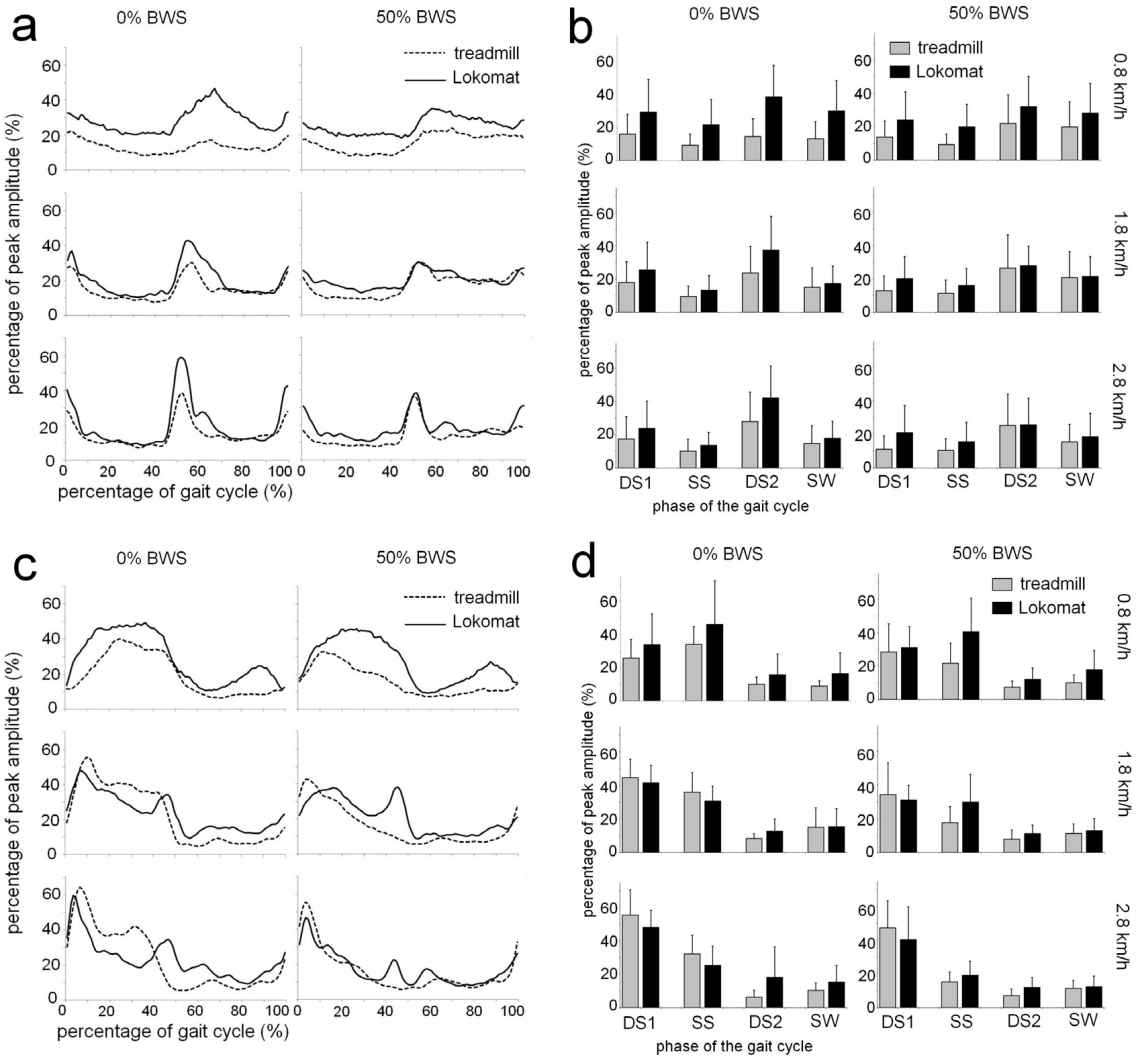
EMG profiles and average muscle activity per gait phase for Erector Spinae and Gluteus Medius. A: Time and amplitude normalized EMG profiles for Erector Spinae (ES) during walking in the Lokomat exoskeleton (solid lines) and during treadmill walking (dotted lines), at 0.8 km/h (top), 1.8 km/h (middle), and 2.8 km/h (bottom), at 0% (left column) and 50% body weight support (BWS; right column). EMG amplitude is expressed as a percentage of peak amplitude recorded over all conditions. B: Average level of ES activity in all walking conditions (see above for further explanation), for four subphases of the gait cycle (DS1: first double support phase; SS: single support phase; DS2: second double support phase; SW: swing phase). C: Time and amplitude normalized EMG profiles for Gluteus Medius (GM). D: Average level of GM activity for four subphases of the gait cycle.

The average EMG profiles and average EMG values (+sd’s) per subphase of the gait cycle for ES are shown in Figures 2a and 2b. During the DS1, the mean difference in EMG amplitude between walking in the exoskeleton and treadmill walking was 9.1% of peak amplitude, corresponding to a significant main effect for the factor Condition.

During the SS phase, a main effect of Condition showed that muscle activity amplitude was increased during exoskeleton walking when compared to treadmill walking (10.0% vs 16.7% of peak amplitude). However, a significant Condition by Speed interaction indicated that this difference between walking conditions depended on treadmill speed. At 0.8 km/h, activity during SS was substantially higher in the exoskeleton than during treadmill walking (20.8% vs 9.3%), but reduced to levels comparable to treadmill walking as speed increased (14.5% vs 10.3% at 2.8 km/h).

For the DS2 phase, a significant Condition by BWS interaction indicated that differences between exoskeleton and treadmill walking were attenuated by providing BWS, with larger differences between walking conditions being observed during full weight bearing (22.0% vs 39.3% for treadmill and exoskeleton, respectively) than when BWS was provided (24.6% vs 28.5%). A significant Speed by BWS interaction indicated that speed effects on ES activity during DS2 were modulated by BWS: whereas during full weight bearing the mean ES activity was increased by 8.5% between 0.8 and 2.8 km/h, similar increases in speed resulted in a decrease of 1.8% in ES activity when 50% BWS was provided.

With regard to the SW phase, a significant Condition by Speed interaction showed that the magnitude of differences between walking conditions depended on treadmill speed. At 0.8 km/h, activity during the SW phase was higher in the exoskeleton than during treadmill walking (29.1% vs 16.5%), but attained levels comparable to treadmill walking when speed increased to 2.8 km/h (18.1% vs 15.1%).

#### Gluteus Medius (GM)

EMG profiles and mean EMG values per gait cycle phase (+sd’s) for GM are presented in Figures 2c and 2d. For the DS1 phase, a significant main effect for Speed indicated that increases in treadmill speed resulted in higher GM amplitudes (29.7% at 0.8 km/h vs 47.9% at 2.8 km/h).

During the SS phase, providing 50% BWS resulted in an decrease in GM activity, compared to full weight bearing conditions (24% vs 34%), which corresponded to a significant main effect of BWS. Also, a significant Condition by Speed interaction revealed that the effects of gait speed on GM activity were different for exoskeleton and treadmill walking. Whereas in the exoskeleton activity decreased form 43.2% at 0.8 km/h to 22.3% at 2.8 km/h, during treadmill walking GM activity was relatively stable over speeds (27.8% at 0.8 km/h vs 23.8% at 2.8 km/h).

#### Biceps Femoris (BF)

**Figure 3:**
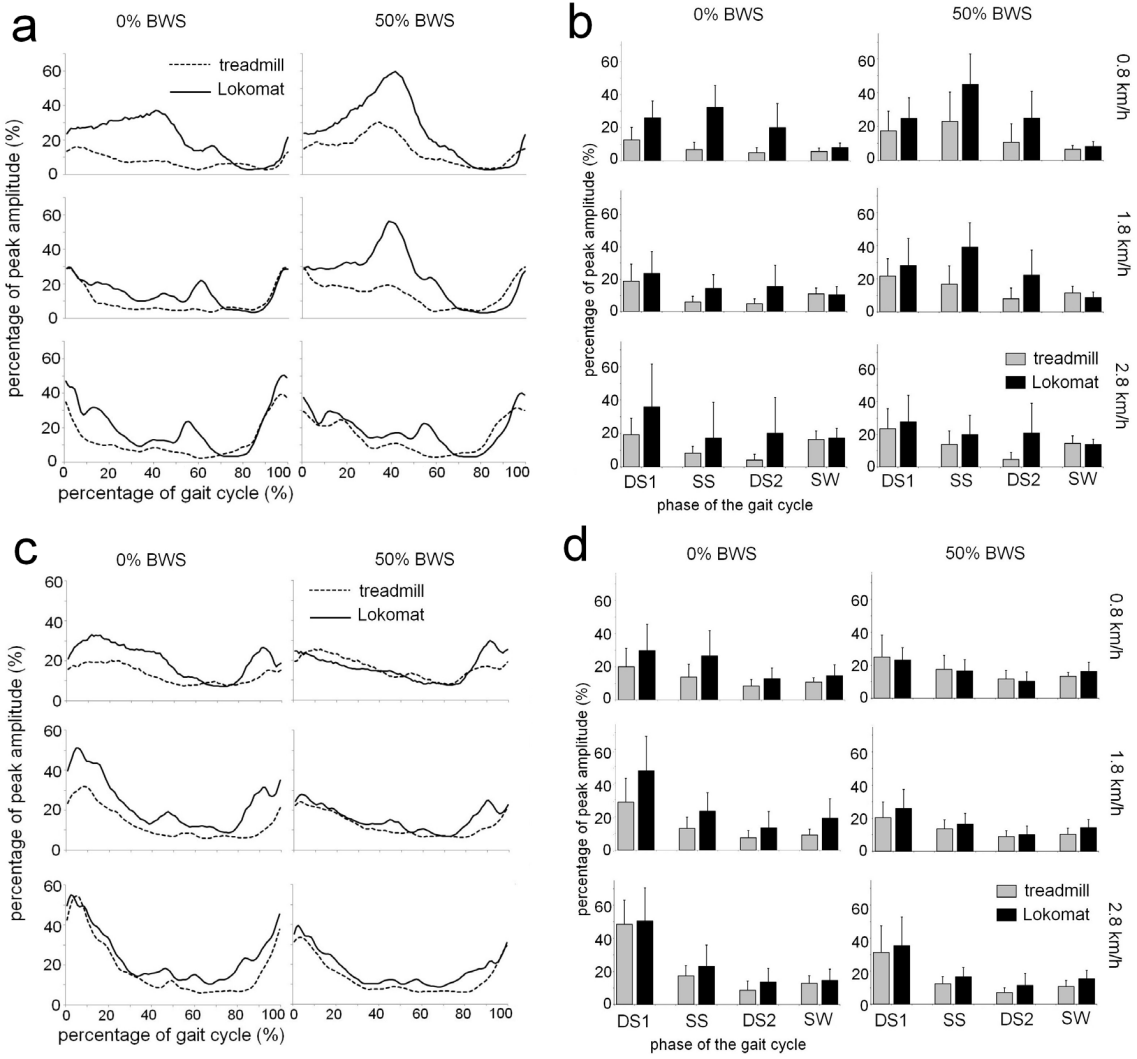
EMG profiles and average muscle activity per gait phase for Biceps Femoris and Vastus Lateralis. Time and amplitude normalized EMG profiles (left column) and the average level of muscle activity for four subphases of the gait cycle (right column) for Biceps Femoris (A+B) and Vastus Lateralis (C+D). See figure 2 for further details.

In Figures 3a and 3b, EMG profiles and mean EMG values per gait cycle phase (+sd’s) are presented for BF. During the SS phase, a significant Condition by Speed interaction indicated that differences between walking conditions depended on treadmill speed. During walking at 0.8 km/h, activity of BF was higher when walking in the exoskeleton then during treadmill walking (38.6% vs 14.9%), and was substantially smaller when walking at higher speeds (18.5% vs 11.1% at 2.8 km/h). Further, a Speed by BWS interaction revealed that the effects of BWS differed for the different levels of gait speed. At 0.8 km/h, BF activity was during SS phase was higher when BWS was provided (33.9%) then under full weight bearing (12.8%), but this difference between weight bearing conditions was less pronounced at higher gait speeds (16.9% vs 12.8% at 2.8 km/h).

During the DS2 phase, BF activity was higher when walking in the exoskeleton than during treadmill walking (20.6% vs 6.2%), as indicated by a main effect of Condition. Finally, during the SW phase, a significant main effect of Speed showed that BF activity increased with speed (7.1% at 0.8 km/h vs 15.5% at 2.8 km/h). However, a Speed by BWS interaction revealed that these speed effects were more pronounced under full weight bearing than when BWS was provided (mean difference between 0.8 and 2.8 km/h 10.0% vs 6.9%).

#### Vastus Lateralis (VL)

Figures 3c and 3d show the mean EMG profiles, and the mean EMG (+sd’s) values per gait cycle phase. A Condition by BWS interaction for VL activity during DS1 showed that the higher amplitude of activity in the exoskeleton (42.5%) compared to treadmill walking (32.3%), were attenuated when 50% BWS was provided (28.1% vs 25.5%). Also, a main effect of Speed showed that VL activity during this phase increased with treadmill speed (24.5% at 0.8 km/h vs 41.4% at 2.8 km/h), but a significant Speed by BWS interaction indicated that the effects of Speed were more outspoken under full weight bearing conditions (average increase between 0.8 and 2.8 km/h of 24.6%), than when BWS was applied (average increase 9.3%).

During the SS phase, a significant interaction of Condition by BWS indicated that the difference between exoskeleton and treadmill walking were significantly smaller when BWS was provided (mean difference 2.0%) than under full weight bearing conditions (9.5%). A similar Condition by BWS interaction was observed during the DS2 phase, where the mean difference between treadmill walking and walking in the exoskeleton was smaller when BWS was provided (1.4%) compared to full weight bearing (5.2%).

During SW, walking in the exoskeleton led to an increase in VL activity compared to treadmill walking (11.2% vs 16.8%), as indicated by a main effect of Condition. However, a significant Condition by BWS interaction revealed that these differences were significantly less pronounced when BWS was provided (7.3% vs 3.9% for 0% and 50% BWS, respectively). Also, an interaction between Speed and BWS showed that speed related increases in VL activity during the SW phase, were seen during full weight bearing (12.7% at 0.8 km/h vs 16.9% at 2.8 km/h), while small decreases were apparent when BWS was supplied (14.8% vs 13.1%).

#### Medial Gastrocnemius (MG)

**Figure 4:**
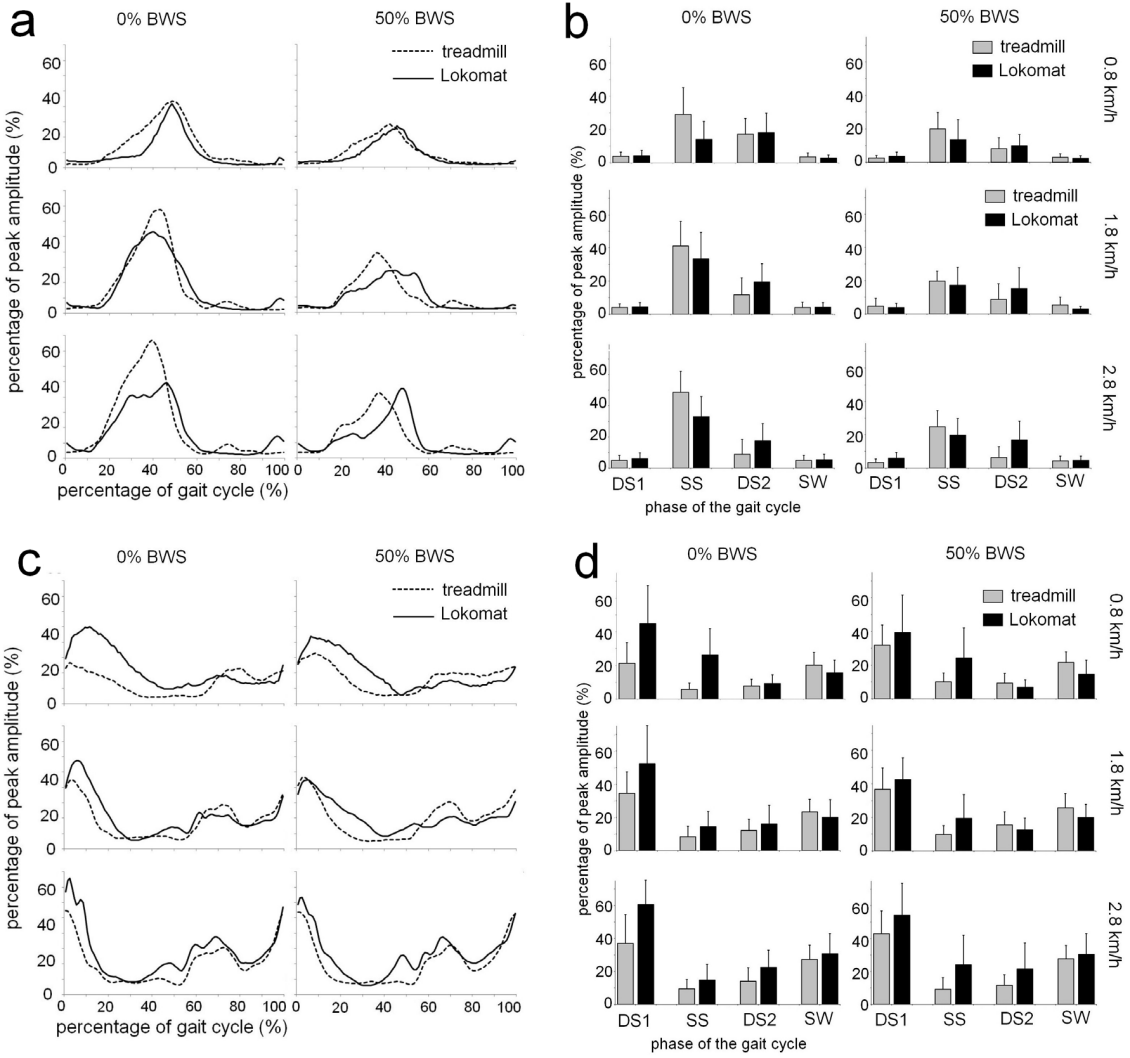
EMG profiles and average muscle activity per gait phase for Gastrocnemius Medialis and Tibialis Anterior. Time and amplitude normalized EMG profiles (left column) and the average level of muscle activity for four subphases of the gait cycle (right column) for Gastrocnemius Medialis (A+B) and Tibialis Anterior (C+D). See figure 2 for further details.

The average EMG profiles and average EMG values (+sd’s) for each of the gait cycle phases for MG are shown in Figures 4a and 4b. For DS1, a significant main effect of Speed revealed that MG activity increased with speed during the DS1 phase (3.6% at 0.8 km/h vs 5.0% at 2.8 km/h). A similar main effect of Speed during the SS phase was detected (19.2% vs. 31.5% at 0.8 and 2.8 km/h, respectively), although the magnitude of this effect depended on BWS conditions, as indicated by a significant Speed by BWS interaction. Speed dependent increases in MG activity were more prominent during full weight bearing (average increase between 0.8 and 2.8 km/h: 19.2%) than when BWS was provided (5.4%).

During the DS2 phase, a Speed by BWS interaction revealed that the effects of Speed depended on whether BWS was provided. A speed dependent decrease of activity was seen during full weight bearing (17.7% vs 13.2% at 0.8 and 2.8 km/h, respectively), while a speed dependent increase was observed when BWS was provided (9.0% vs 11.6%).

Finally, during the SW phase a activity of MG increased with speed (3.0% at 0.8 km/h vs 4.8% at 2.8 km/h), as revealed by a significant main effect of Speed.

#### Tibialis Anterior (TA)

Average EMG profiles and the average normalized amplitudes (+ sd’s) of TA activity for the four different gait phases are depicted in Figures 4c and 4d. During the DS1 phase, a main effect of Condition showed that levels of activity where higher in the exoskeleton than during treadmill walking (49.0% vs 34.0%), although this effect was attenuated when BWS was provided (mean differences between exoskeleton and treadmill 21.7% and 8.3% for 0% and 50% BWS, respectively), as indicated by a Condition by BWS interaction. Also during DS1, a main effect of Speed was found (average TA activity 34.3% and 48.7% at 0.8 km/h and 2.8 km/h).

During the SS phase, TA activity was higher during exoskeleton walking than during treadmill walking (19.1% vs 8.8%), as revealed by a main effect of Condition. However, a significant Condition by Speed interaction indicated that differences between walking conditions depended on treadmill speed. The difference in TA activity during SS was larger at 0.8 km/h (average difference 17.3%) than at 2.8 km/h (5.8%).

A significant main effect of Speed indicated that the amplitude of TA activity during DS2 depended on treadmill speed (8.3% vs 17.4% at 0.8 and 2.8 km/h, respectively). However, these speed dependent increases were more outspoken during exoskeleton walking than during treadmill walking (average increase of 14.0% vs 4.3%), as revealed by a significant Condition by Speed interaction. Similar effects were apparent during the SW phase: a main effect of Speed signified an increase of activity with speed (18.0% vs 29.0% at 0.8 and 2.8 km/h, respectively), but this effect was larger during exoskeleton walking, compared to treadmill walking (average increase 15.5% vs 6.6%) resulting in a significant Condition by Speed interaction.

## Discussion

To fully exploit the potential of RAGT and aid the development of purposeful training protocols for this training environment, it is important to understand the respective effects of the exoskeleton, treadmill speed, and BWS, as well as their mutual interactions, on locomotor control. Therefore, the present study assessed temporal step parameters and muscle activity during walking in the Lokomat exoskeleton and during unrestrained treadmill walking, while gait speed and BWS support were varied systematically.

### Walking in the exoskeleton alters the temporal structure of the stepping pattern

In agreement with previous studies, the present results show that during treadmill walking the relative duration of the SS phase increased, and the relative duration of the DS phase decreased with gait speed [25]–[27]. However, the magnitude of these speed effects strongly depended on walking condition, since speed dependent modulations of step phase durations that were observed during treadmill walking were virtually absent when participants walked in the exoskeleton. As a result of the interacting effects of treadmill speed and walking condition, differences in the temporal structure of the stepping pattern between exoskeleton and treadmill walking were most outspoken at slower speeds, and became more similar as treadmill speed increased. During unrestrained walking, the temporal structure of the stepping pattern, and its modulation by speed, is determined mainly by passive properties of the swinging leg, most notably its length and its mass or inertia [8]. Walking in the exoskeleton changes the inertial properties of the leg, both through the added mass of the exoskeleton and the control-response inertia that results from interaction forces between the leg and the exoskeleton [18]. In the absence of the supportive torques that normally guide the leg movements during training, the inertia of the exoskeleton is only partially compensated for by the cooperative Path Control algorithm that is used to drive the segments of the Lokomat [15]. The observed prolongation of the SS phase may be related to active efforts to overcome this increased resistance to limb acceleration, since adding mass to the leg is known to prolong swing times [7], [9], [28].

In line with previous research [13], [29], providing 50% BWS resulted in a decreased duration of the DS phases and a concomitant increase in SS duration. The magnitude of these effects strongly depended on gait condition since the temporal structure of stepping in the exoskeleton was more similar to treadmill walking when 50% BWS was provided than under full weight bearing conditions. However, it must be noted that BWS related changes in step phase durations in the exoskeleton did not reflect a normalization of the stepping pattern, as these effects were mostly due to abnormal durations during BWS walking outside the exoskeleton (i.e. shortening of DS, lengthening of SS). These observed changes in the duration of step phases are possibly related to decreased balance constraints when weight support is provided [13], resulting in a longer time spent in the unipedal phase. In addition, because leg-loading is known to suppress swing initiation [30], reduction in loading information due to BWS may cue an early initiation of the swing phase, as was observed in the present data.

To summarize, changes in the temporal structure of stepping induced by the exoskeleton can to be minimized if BWS is provided and treadmill speed is increased.

### Differences in muscle activity between exoskeleton and treadmill walking depend on treadmill speed and BWS

In a recent study, Gizzi and co-workers [16] showed that the modular organization of neuromuscular activity that is typical of bipedal human locomotion, is similar between overground walking and walking in the Lokomat exoskeleton, and is largely unaffected by changes in gait speed and BWS. Overall, the present results confirm these findings, showing that the gross temporal patterning of muscle activity is maintained over conditions, and that variations in speed, BWS, and walking condition (treadmill vs exoskeleton) do not give rise to the emergence of new bursts of muscle activity or a profound restructuring of neuromuscular patterns. However, the results also demonstrate that at the level of individual muscles, changes in gait conditions cause a local re-scaling of muscle output amplitude.

A more detailed analysis of the activity patterns showed that at the level of individual muscles (ES, BF, VL, and TA), the overall levels of muscle activity were higher when walking in the exoskeleton than during treadmill walking, in particular during the stance phase. Arguably, this increase in activity may be related to efforts to overcome the inertial mass of the exoskeleton. If this was the case then it could be expected that differences in the amplitude of muscle activity between exoskeleton and treadmill walking increase with speed, since the larger segmental accelerations associated with higher speeds would require more muscle force output to overcome exoskeleton inertia. However, the results showed that for several muscles (ES, GM, BF, and TA) increases in the speed of exoskeleton walking were associated with reductions in muscle output amplitude, so that activity became more similar between treadmill and exoskeleton walking a higher speeds. Similarly, a BWS by Condition was apparent for stance activity in ES, VL and TA as activity in the exoskeleton was higher than during treadmill walking under full weight bearing conditions, but attained comparable levels when 50% of the participants’ body weight was supported.

Apparently, aberrations in muscle activity and temporal step characteristics emerged in the same conditions (i.e. abnormal behaviour in the exoskeleton at slow speeds and/or when no BWS was applied, near normal behaviour at 2.8 km/h and/or when BWS was provided), suggesting that the observed modifications in muscle activity and step control are functionally related. More specifically, prolongation of the SS phase may have enforced modifications in neuromuscular control to accommodate the altered task constraints implied in changing the natural relationship between gait speed and step phase durations. Indeed, studies on the intentional modification of the preferred step frequency–to-amplitude relationship have demonstrated that such alterations in the temporal step pattern are accompanied by a reorganization of the underlying muscle activity of e.g. the quadriceps and hamstrings [31]–[32].

Alternatively, because during the unipedal phase the head-arm-trunk segment is supported by one leg, prolongation of the SS phase may have imposed increased demands on lateral stability and body support, necessitating additional muscular effort. For instance, because ES aids trunk stabilization during ipsilateral and contralateral foot landing [11], [33], the observed increases in ES activity in the exoskeleton may have aided lateral stability, a neuromuscular strategy that is adopted by hemiparetic stroke patients to reduce body sway during unaided walking [34]. Likewise, the increased TA stance activity that was found in the exoskeleton at the slowest speed, may have served to generate stabilizing torques around the ankle during the prolonged unipedal phase [35], [36]. In line with the present results, these adaptations in neuromuscular control are likely to be attenuated as treadmill speed increases and less emphasis is put on mediolateral balance control (e.g. due to larger center of mass excursions at slow speeds [37]), or when BWS diminishes demands on balance and support.

### Support of body weight reduces muscle activity and attenuates speed effects

Although the observed EMG patterns displayed the increases in amplitude that typically accompany increases in gait speed [27], [38], [39], for some muscles (ES, VL, and MG) speed effects where attenuated when BWS was provided. During unsupported walking, increases in gait speed are accompanied by larger impact forces at foot contact [40] that need to be actively counteracted by generating additional force output in the appropriate muscle groups. Consequently, reductions in impact forces through body unloading are likely to decrease the need for larger force outputs, resulting in a diminished modulation of EMG amplitude by speed in muscles concerned with the control of foot impact (ES) and weight acceptance (VL). With regard to MG, a recent investigation has shown that speed related increases in the stance activity of this muscle are primarily related to speed dependent increases in support demands rather than to propulsive effort [41]. Therefore, it seems logical that reductions in support demands through BWS result in an attenuation of speed effects that are observed in MG in the full weight bearing conditions.

In accordance with previous research [13], [27], leg unloading through BWS lead to a reduction of EMG amplitude during the stance phase in ES, GM, VL, and MG. In contrast, the amplitude of muscle activity was increased in BF when BWS was provided, in particular at lower speeds. The support of body weight diminishes task demands related to weight transfer and as such can be expected to reduce muscular effort to control foot impact (ES), weight acceptance (VL) and weight transfer (GM). With respect to plantarflexor activity, studies on animals [42] and humans [3] have shown that load information provides an important sensory cue that drives ankle flexor activity. Therefore, reductions in loading brought about by BWS are likely to be accompanied by reductions in MG amplitude, as was the case in the present study. Diminished activity in ankle plantarflexors may also explain the increases in BF activity in response to BWS, since hip extension may have been used to compensate for the reduced activity of ankle extensors in providing support and forward trunk propulsion during stance [43]. The observed similarity in the shape of BF and MG bursts during stance, particularly at slow speeds, seems to provide support for this idea.

### Clinical implications

For a purposeful exploitation of training parameters in RAGT, it is essential to understand how parameter settings affect locomotor control. The results of this study show that walking in the Lokomat exoskeleton without movement guidance may alter step regulation and the neuromuscular control of walking, but that the nature and magnitude of these effects depend on treadmill speed and BWS. The results also demonstrated that the effects of treadmill speed on muscle activity and temporal step control depend on the amount of BWS that is provided. Therefore, the present results emphasize that (1) the purposeful setting of training parameters in RAGT should consider the combined effects of treadmill speed and BWS in their interactive context, and (2) that when possible, training at higher speeds with low levels of BWS should be favored in training conditions that target a normative neuromuscular control of gait.

A key feature of RAGT is the possibility to generate supportive torques and provide guidance of leg movements by the exoskeleton [15]. Although the effects of movement guidance were not considered in this study, the result are clinically relevant since training is often aimed at reducing the level of guidance as training progresses, thereby implicitly assuming that the minimization of guidance creates training conditions that resemble the set of constraints that typify natural walking. The present findings suggest that this only holds true when extremely slow speeds are avoided and levels of BWS are kept at a minimum. These considerations should also be taken into account when asymmetrical support is considered, e.g. for the training of hemiparetic patients, and guidance is restricted to the affected leg while the unaffected leg is allowed to walk in ‘free walking’ mode. It is important to note that, although reductions of guidance force can be an effective means to promote active participation of patients, setting guidance levels to zero, as was done in the present study, may not be representative of common training conditions [44]. Therefore, it is important that future research efforts will elaborate on the present results, uncovering the interrelationships between BWS, treadmill speed and movement guidance. In addition, a logical extension of the present work is to systematically assess these effects in the patient populations that are targeted in RAGT like stroke, spinal cord injury and cerebral palsy, and to establish how training parameters interact with the known neuromuscular abnormalities in these groups [45]–[47].

### Conclusion

The main aim of the present study was to determine the combined effects of BWS and gait speed on gait related muscle activity, and compare these between regular treadmill walking and walking in the Lokomat exoskeleton. The results show that walking in the Lokomat exoskeleton alters the temporal regulation of steps as well as the neuromuscular control of walking, and that the nature and magnitude of these effects depend on complex interactions with gait speed and BWS. Based on these finding, it can be suggested that, if normative gait patterns are desired, very low speeds and high levels of BWS should be avoided when possible.
